# An overestimation of the prevalence of ASD among psychiatric patients

**DOI:** 10.1007/s10803-022-05568-1

**Published:** 2022-04-28

**Authors:** Susanne Bejerot, Lena Nylander

**Affiliations:** 1 School of Medical Sciences, Örebro University, Örebro, Sweden; 2 Department of Clinical Sciences, Lund University, Lund, Sweden

To our surprise a study by Nyrenius et al. (2022) suggests that ASD is prevalent among psychiatric non-psychotic outpatients based on a two-hour interview, including the M.I.N.I., with 48 individuals of whom 26 were deemed to have ASD. To begin with, the study suffers from a very low participation rate at all sampling phases, from both study subjects and their parents.

Nyrenius et al. based the ASD diagnoses on a 14 item self-rated screening instrument (RAADS-14 Screen) (Eriksson et al., 2013), the short 20 item Asperger Syndrome (and high-functioning autism) Diagnostic Interview (ASDI) performed with the patient (Gillberg et al., 2001), and a parent questionnaire on neurodevelopmental symptoms (5–15) (Kadesjö et al., 2004). There are no reliability (test–retest) data for the screening tool and the other instruments. The specificity of the RAADS, as quoted out of a different study, is low (67%) and suggests that false positives are likely a pervasive issue in that study.

The ASDI is intended for use with informants who know the individual very well–and who knew them well when they were children—this procedure was not applied in the Nyrenius study. Neither is the 5–15 reliable for retrospective use in adults because parents tend to forget how their child behaved years before (Lugnegård & Bejerot, 2019). Moreover, in the Nyrenius’ study the 20 parents who did fill out the 5–15 were asked, incomprehensibly, to report their child’s neurodevelopmental symptoms at age 17, when the DSM-5 is stating that symptoms should be present in “early childhood”. It would have been more adequate and possible to ask about symptoms at age 5. There is no reason to not add other sources of information such as review of medical records and/or obtaining data on the developmental history in the first 5 years.

Differential diagnostic considerations are not accounted for, although important in this setting since the instruments used have low specificity, especially in adults. Co-occurring psychiatric diagnoses are not described, which is also a limitation.

The study arrives at a conclusion that the minimum prevalence of ASD in the studied group was 18.9%, assuming a similar prevalence of ASD in the non-responder group as in the responders. However, if the minimum prevalence is calculated in the same way as was done in a previous Swedish study (Nylander & Gillberg 2001), namely the number of confirmed cases (n = 26) out of the entire screened group (n = 304) the resulting minimum prevalence would be no more than 8.6% which is still considerably larger than the population prevalence estimations. It is also larger than the 1.4% that was found by Nylander & Gillberg, which is not unexpected since the diagnostic criteria have changed since 2001. In the Nylander et al. study the patients (95%—n = 1323—of all out-patients in a certain area), were screened by psychiatric staff for suspected autistic behaviors. It is true, as Nyrenius et al. point out, that the psychiatric staff in 2001 were not familiar with the concept of autism, but it is debatable if unselected psychiatric patients, filling out the self-report RAADS-14, are better judges of autistic symptoms than psychiatric personnel.

Other problems with this study are that the "validation" study is poorly described, performed in an unexplained manner, on a very small sample (n = 5), and little confidence can be attached to this exercise, and that there is no 95% CI anywhere. Another limitation is presumably a selection bias, i.e., patients who chose to participate in the study may represent individuals with a high load of autistic traits, and subsequently wanting to be assessed for ASD in order to be diagnosed, whereas others may have lacked interest in this extra diagnostic procedure. Finally, Nyrenius et al. support their findings of a high prevalence of ASD by comparing them to the prevalence rates reported among depressive out-patients in Japan (Takara & Kondo, 2014). However, the Japanese study suffers from methodological issues, as reflected by unexpected findings, e.g. 63% of the participants who were diagnosed with ASD had never experienced any interpersonal frictions or been bullied in school, their educational level did not differ from non-autistic participants and 36% were married.

It is certainly important to keep in mind that a few of the individuals who seek psychiatric treatment may have undiagnosed autism or other information processing difficulties, and to adjust psychiatric services to accommodate these patients in a way that ensures good treatment for everyone. However, people with autism are not helped by inflated numbers based on assumptions rather than facts. Research on autism should favor production of knowledge (Mottron, 2021).
